# Inflammatory Breast Cancer: A Distinct Clinicopathological Entity Transcending Histological Distinction

**DOI:** 10.1371/journal.pone.0145534

**Published:** 2016-01-11

**Authors:** K. Raghav, J. T. French, N. T. Ueno, X. Lei, S. Krishnamurthy, J. M. Reuben, V. Valero, N. K. Ibrahim

**Affiliations:** 1 Department of Gastrointestinal Medical Oncology, Divison of Cancer Medicine, The University of Texas MD Anderson Cancer Center, Houston, Texas, United States of America; 2 Division of Cancer Medicine, The University of Texas MD Anderson Cancer Center, Houston, Texas, United States of America; 3 Department of Breast Medical Oncology, Divison of Cancer Medicine, The University of Texas MD Anderson Cancer Center, Houston, Texas, United States of America; 4 Department of Morgan Welch Inflammatory Breast Cancer Research Program and Clinic, Divison of Cancer Medicine, The University of Texas MD Anderson Cancer Center, Houston, Texas, United States of America; 5 Department of Biostatistics, Divison of Cancer Medicine, The University of Texas MD Anderson Cancer Center, Houston, Texas, United States of America; 6 Department of Pathology, Divison of Cancer Medicine, The University of Texas MD Anderson Cancer Center, Houston, Texas, United States of America; 7 Department of Hematopathology, Divison of Cancer Medicine, The University of Texas MD Anderson Cancer Center, Houston, Texas, United States of America; Wayne State University School of Medicine, UNITED STATES

## Abstract

**Introduction:**

Although well recognized in breast oncology literature, histologic subtypes have not been previously described in inflammatory breast cancer (IBC). The purpose of this study was to describe lobular subtype in IBC and assess the impact of histology on patient outcomes.

**Methods:**

We performed a retrospective analysis of 659 IBC patients at MD Anderson Cancer Center between January 1984 and December 2009. Patients with Invasive Lobular, Mixed Invasive Ductal and Lobular, or Invasive Ducal Carcinomas (ILC, MIC, IDC, respectively) comprise the subject of this report. Patient characteristics and survival estimates were compared by using chi-square test and Kaplan-Meier method with log-rank statistic. Cox proportional hazards models were fit to determine association of histology with outcomes after adjustment for other characteristics.

**Results:**

A total of 30, 37, and 592 patients were seen to have invasive lobular, mixed, or ductal histology, respectively. Grade 3 tumors were more common in the ductal group (78%) than in the lobular (60%) or mixed (61%) group (*P* = 0.01). The 3-year overall survival rates were 68%, 64%, and 62% in the lobular, mixed, and ductal groups, respectively (*P* = 0.68). After adjustment, histology did not have a significant effect on death in the lobular group (HR = 0.70, 95% confidence interval [CI]: 0.26–1.94; *P* = 0.50) or mixed group (HR = 0.53, 95% CI: 0.25–1.13; *P* = 0.10) compared with the ductal group.

**Conclusion:**

In this cohort of IBC patients, lobular histology was seen in 4.5% cases. Histology does not appear to have a significant effect on survival outcomes in IBC patients, unlike in patients with non-inflammatory breast cancer (n-IBC), indicating the distinct biological behavior of the IBC phenotype.

## Introduction

Inflammatory breast cancer (IBC) is a rare and aggressive form of breast cancer[1]. This phenotype is characterized clinically by acute inflammatory changes of the breast presenting, within ≤ 3 months, diffuse erythema and edema, with/without palpable mass, and biopsy proven invasive carcinoma of the breast; pathological infiltration of dermal lymphatics by tumor emboli may not be pathognomic, however[2]. IBC accounts for 1% to 2% of all invasive breast cancers[3]. It is characterized by a higher risk of early recurrence, distant metastases, and metastases to the central nervous system compared with non-inflammatory locally advanced breast cancer[4, 5]. In addition, IBC has poorer survival rates when looking at all stages (5-year survival 40% vs. 87%), compared to n-IBC, despite the similar multidisciplinary care given for both diseases[3, 4]. Concerted efforts toward the characterization and understanding the clinical and biology features of this rare aggressive malignancy is critical to improving patient outcomes.

Although IBC is well defined clinically, it is not however, been described as having a distinct histology[6, 7]; the presence of neoplastic dermal lymphatic emboli is neither necessary nor sufficient for its diagnosis[7]. However, in most cases, the tumor is often characterized as ductal type with the emboli composed of pleomorphic tumor cells with high nuclear grade[8]. Nevertheless, IBC is being increasingly recognized as a distinct molecular breast cancer subtype[9].

In n-IBC, histology subtypes dictate clinical presentation and the natural history of the disease[10]. Lobular histology is seen in about 8% of invasive breast cancer and is characterized by a unique clinicopathological profile that is distinct from invasive ductal carcinoma (IDC)[10]. Multiple studies show patients with invasive lobular carcinoma (ILC) have better long-term outcomes than patients with IDC[11, 12]. This histologic distinction between ILC and IDC is also predictive of response to therapy, with ILC characterized by lower rates of pathologic response to chemotherapy[12].

Non inflammatory ILC and IDC have distinctive molecular profiles, both at a genomic and proteomic level, further supporting the role of histology in dictating biological behavior[13, 14]. Currently, treatment recommendations do not differ significantly between lobular and ductal histology[15]. Although ILC is recognized as a distinct entity, there has been no study describing the role of histologic distinction between lobular and ductal subtypes in IBC. Whether it represents a heterogeneous group like n-IBC or is strictly a homogeneous entity is not clearly understood. Recognizing the importance of distinguishing among histologic subtypes of n-IBC, we aim at exploring the significance of such histologic subtyping in the clinical presentation and outcomes that may be present or associated with IBC, with a special emphasis on lobular IBC.

## Methods

### Patient population

We performed a retrospective review using the Department of Breast Medical Oncology electronic database at University of Texas MD Anderson Cancer Center, Houston, Texas. This study was reviewed and approved by the University of Texas MD Anderson Cancer Center Institutional Review Board. Patient records and information were anonymized and de-identified prior to analysis. Written informed consent was obtained by participants. We identified 659 patients with IBC who had been evaluated and treated at MD Anderson between January 1, 1984 and December 31, 2009. Wherever required, missing data were obtained by review of individual medical records.

The clinicopathological factors of interest included age at diagnosis, ethnicity, menopausal status, tumor grade, histopathology, estrogen receptor (ER) status, progesterone receptor (PR) status, human epidermal growth factor receptor 2 (HER2) status, lymphatic and vascular involvement, sites of metastases (grouped as brain, visceral, or nonvisceral metastases), and TNM stage. ER and PR status was determined by immunohistochemical analysis and was considered positive if >10% of cells were stained for ER and/or PR. HER2 status was assessed by immunohistochemical analysis or fluorescence in situ hybridization (FISH) and was considered positive if >30% of cells were stained (IHC 3+) or if the HER2/centromere enumerator probe (CEP) ratio on FISH was ≥ 2.0. Breast pathologists at MD Anderson reviewed all pathological findings. Treatment characteristics included adjuvant radiation, hormonal therapy, adjuvant/neoadjuvant chemotherapy, adjuvant/neoadjuvant anthracycline and taxane use, and definitive surgery (defined as either lumpectomy or mastectomy).

### Methodology

Patients were divided in 3 groups: ILC, MIC or IDC. The primary end point was overall survival (OS), measured from the date of diagnosis to the date of death. Patients were classified as either metastatic (M1) or non-metastatic (M0) disease. The time to first progression (TTP1) of the M1 group was measured from the date of the first documented metastasis to the date of first progression. On the other hand, M0 group distant metastasis-free survival (DMFS) was measured from the date of diagnosis to the date of first documented distant metastasis. Recurrence-free survival (RFS) was measured from the date of diagnosis to the date of first documented local or distant recurrence, and death. Patients not experiencing the relevant end point were censored at the last follow-up.

### Statistical analysis

Patient characteristics were compared among the three groups with the chi-square test or Fisher’s exact test, as appropriate. The Kaplan-Meier product limit method was used to estimate the survival outcomes of all patients’ groups. Groups were then compared with the log-rank statistic. Cox proportional hazards models were fit to determine the association of histology with survival outcome after adjustment for other patient and disease characteristics. Variables adjusted included age (>60, ≤60), race (black, non-black), hormone status (positive, negative), HER2 status (positive, negative), grade (I/II, III), stage (M0, M1), lymphovascular invasion (positive/ negative), number of metastasis (continuous), brain metastasis (yes, no), visceral metastasis (yes, no), and adjuvant radiation (yes, no). Subset analyses of patients with M1 or M0 tumors were conducted. *P* values of less than 0.05 were considered statistically significant, and all tests were two-sided. Statistical analyses were conducted with use of SAS 9.2 (SAS Institute Inc., Cary, NC) and S-Plus 7.0 (Insightful Corporation, Seattle, WA).

## Results

### Baseline characteristics

Of patients with IBC (n = 659), 30 patients (4.6%) were identified with ILC, 37 (5.6%) with MIC, and 592 (89.8%) with IDC. The median age at diagnosis was 53.5, 52, and 49 years in the lobular, mixed, and ductal groups, respectively (range 22 to 87 years); 78% of IDC patients p had grade 3 tumors compared with 60% ILC and 61% in the MIC groups (*P* = 0.01). Stage IV patients accounted for 23% in the IDC group, compared with 47% and 35% in the ILC and MIC group, respectively (*P* = 0.04). Neoadjuvant chemotherapy was given to 76% of patients with IDC, compared with 57% in the lobular and 62% in the mixed groups (*P* = 0.01). All other patient and clinical characteristics including age, menopausal status, race, tumor subtype (ER/PR/HER2), nodal status, lymphatic and vascular involvement, number and sites of metastases, definitive surgery, and adjuvant therapy were similar among the three histologic groups (Table 1).

**Table 1:** Patient demographics, tumor characteristics, and therapy.

	Ductal (*n* = 592)		Lobular (*n* = 30)		Mixed (*n* = 37)		*P* value
	*n*	Percent	*n*	Percent	*n*	Percent	
Age, median	49		53.5		52		
Age (yr)							
≤60	486	82.1	22	73.3	30	81.1	
>60	106	17.9	8	26.7	7	18.9	0.48
Menopausal Status							
Premenopausal	281	48.0	9	30.0	15	41.7	
Postmenopausal	304	52.0	21	70.0	21	58.3	0.13
Race							
Non-Black	523	88.3	28	93.3	33	89.2	
Black	69	11.7	2	6.7	4	10.8	0.82
Subtype							
Hormone-positive	191	38.1	13	56.5	15	41.7	
HER2-positive	176	35.1	7	30.4	13	36.1	
Triple-negative	134	26.7	3	13.0	8	22.2	0.45
Grade							
1 or 2	124	22.2	10	40.0	14	38.9	
3	435	77.8	15	60.0	22	61.1	0.01
Clinical stage							
IIIB	346	58.6	11	36.7	18	48.6	
IIIC	106	18.0	5	16.7	6	16.2	
IV	138	23.4	14	46.7	13	35.1	0.04
Lymphovascular invasion							
Negative	177	34.4	11	44.0	12	41.4	
Positive	337	65.6	14	56.0	17	58.6	0.48
Number of metastases							
0	220	37.2	6	20.0	14	37.8	
1	198	33.4	15	50.0	13	35.1	
2+	174	29.4	9	30.0	10	27.0	0.32
Surgery							
Lumpectomy	5	1.0	0	0	0	0.0	
Mastectomy	469	95.9	24	100	27	93.1	
No definitive surgery	15	3.1	0	0	2	6.9	0.62
Neoadjuvant chemotherapy							
No	142	24.0	13	43.3	14	37.8	
Yes	450	76.0	17	56.7	23	62.2	0.01
Neoadjuvant chemotherapy response							
Complete/Partial	289	67.2	10	62.5	14	63.6	
Stable/Progressive	141	32.8	6	37.5	8	36.4	0.88
Adjuvant chemotherapy							
No	373	63.0	18	60.0	26	70.3	
Yes	219	37.0	12	40.0	11	29.7	0.63
Adjuvant radiation therapy							
No	247	41.7	17	56.7	15	40.5	
Yes	345	58.3	13	43.3	22	59.5	0.26

### Overall survival

**Fig 1:**
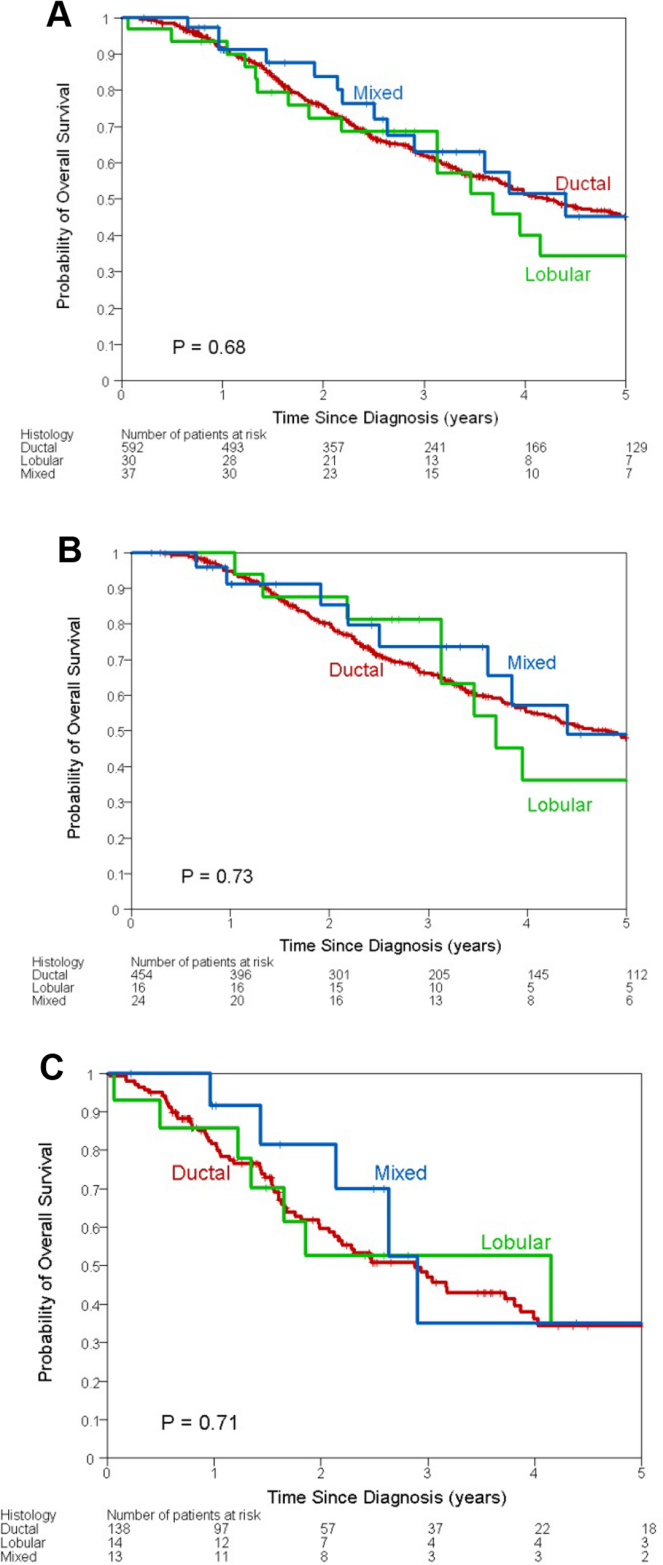
Kaplan-Meier curves of overall survival by histology in (A) all patients, (B) patients with non-metastatic disease, and (C) patients with metastatic disease.

Median follow-up time of all patients was 29 months (range 0 to 244 months). 15 deaths were counted in the lobular group, 13 in the mixed group and 291 in the ductal group. The 3-year OS for the entire cohort was 62% (95% CI: 58%-66%). The 3-year OS was 68% in the lobular group, 64% in the mixed group, and 62% in the ductal group (*P* = 0.68) (Fig 1A). The OS estimates with 95% CI by patient and tumor characteristics are listed in Table 2. Univariate analyses revealed that in all patients, black race, ER/PR-negative tumors, HER2-negative tumors, lymphovascular invasion, multiple sites of metastases, and absence of adjuvant radiation were associated with increased risk of death (Table 2).

**Table 2:** Overall survival estimates by patient and clinical characteristics among all patients.

	Patients	Events	3-year overall survival estimate (95% CI)	*P* value
Total	659	319	0.62 (0.58, 0.66)	
Histology				
Ductal	592	291	0.62 (0.57, 0.66)	
Lobular	30	15	0.68 (0.47, 0.82)	
Mixed	37	13	0.64 (0.43, 0.79)	0.68
Age (yr)				
≤60	538	254	0.65 (0.6, 0.69)	
>60	121	65	0.49(0.39, 0.59)	0.001
Race				
Non-Black	584	269	0.65(0.61, 0.69)	
Black	75	50	0.41 (0.29, 0.53)	<0.001
Subtype				
Hormone-positive	219	82	0.73 (0.66, 0.79)	
HER2-positive	196	79	0.7 (0.62, 0.76)	
Triple-negative	145	93	0.33 (0.24, 0.41)	<0.001
Grade				
I or II	148	58	0.74 (0.65, 0.81)	
III	472	238	0.58 (0.53, 0.63)	0.006
Clinical M stage				
Non-metastatic (M0)	494	235	0.67 (0.62, 0.71)	
Metastatic (M1)	165	84	0.47 (0.38, 0.56)	<0.001
Lymphovascular invasion				
Negative	200	67	0.77 (0.69, 0.82)	
Positive	368	189	0.62 (0.56, 0.67)	<0.001
Number of metastases				
0	240	39	0.85 (0.79, 0.9)	
1	226	144	0.57 (0.5, 0.64)	
2+	193	136	0.44 (0.36, 0.51)	<0.001
Brain, meninges, spinal cord metastasis				
No	617	284	0.65 (0.61, 0.69)	
Yes	42	35	0.25 (0.13, 0.4)	<0.001
Visceral metastasis				
No	461	178	0.71 (0.66, 0.75)	
Yes	198	141	0.44 (0.36, 0.51)	<0.001
Neoadjuvant chemotherapy				
No	169	86	0.51 (0.42, 0.59)	
Yes	490	233	0.66 (0.61, 0.7)	<0.001
Adjuvant radiation therapy				
No	279	158	0.43 (0.36, 0.5)	
Yes	380	161	0.75 (0.7, 0.79)	<0.001

The presence of visceral and brain metastasis was also associated with poor survival (*P* < 0.01). After adjustment for age, race, hormonal status, HER2 status, grade, stage, lymphovascular invasion, brain metastasis, visceral metastasis, and adjuvant radiation, histology did not have a significant effect on OS in the lobular group (HR = 0.70, 95% CI: 0.26–1.94; *P* = 0.50) or mixed group (HR = 0.53, 95% CI: 0.25–1.13; *P* = 0.10) compared with patients in the ductal group (Table 3).

**Table 3:** Cox proportional hazards models for overall survival among patients.

	All patients			M0 patients			M1 patients		
	HR	95% CI	*P* value	HR	95% CI	*P* value	HR	95% CI	*P* value
Lobular vs. Ductal	0.70	0.26–1.94	0.500	0.59	0.14–2.41	0.460	1.20	0.52–2.75	0.660
Mixed vs. Ductal	0.53	0.25–1.13	0.100	0.58	0.25–1.31	0.190	0.91	0.35–2.34	0.840
Age: > 60 vs. ≤ 60	1.36	0.92–2.01	0.120	1.52	1.01–2.29	0.045	1.01	0.59–1.74	0.970
Race: Black vs. Non-black	1.79	1.13–2.84	0.013	1.49	0.89–2.51	0.130	1.75	1.01–3.02	0.046
Hormone status: Positive vs. Negative	0.43	0.31–0.59	<0.001	0.39	0.27–0.55	<0.001	0.32	0.19–0.55	<0.001
HER2 status: Positive vs. Negative	0.48	0.34–0.66	<0.001	0.49	0.35–0.7	<0.001	0.35	0.18–0.66	0.001
Grade: 3 vs. 1/2	1.30	0.86–1.96	0.220						
Clinical M stage: M1 vs. M0	1.00	0.63–1.57	0.990						
Lymphovascular invasion: Yes vs. No	1.70	1.20–2.40	0.003	2.01	1.4–2.89	0.001			
Number of metastases (continuous)	1.20	1.08–1.35	0.001				1.14	0.95–1.36	0.150
Brain metastasis: Yes vs. No	3.58	2.27–5.67	<0.001				4.72	1.36–16.4	0.015
Visceral metastasis: Yes vs. No	1.87	1.30–2.67	<0.001				1.52	0.85–2.72	0.160
Adjuvant radiation therapy: Yes vs. No	0.64	0.44–0.94	0.021	0.50	0.35–0.73	<0.001			

### Outcomes in patients with nonmetastatic disease

Among the 494 patients with M0 tumors, 263 had distant metastases and 278 had either local or distant disease recurrence. The 3-year DMFS and RFS estimates were 0.48 (95% CI: 0.43–0.52) and 0.43 (95% CI: 0.38–0.48), respectively (S1 Table). Histology did not significantly impact either DMFS (*P* = 0.77) or RFS (*P* = 0.65). After multivariable adjustment for age, race, hormone status, HER2 status, lymphovascular invasion, and adjuvant radiation, histology did not have a significant effect on DMFS in the lobular group (HR = 1.62, 95% CI: 0.75–3.51; *P* = 0.22) or mixed group (HR = 0.84, 95% CI: 0.43–1.65; *P* = 0.62) compared with the ductal group (S2 Table). Similarly, histology did not have a significant effect on RFS in the lobular group (HR = 1.39, 95% CI: 0.64–3.00; *P* = 0.40) or mixed group (HR = 0.86, 95% CI: 0.45–1.63; *P* = 0.64) compared with the ductal group (S2 Table).

There were 235 deaths in the M0 group. The 3-year OS estimate was 0.67 (95% CI: 0.62–0.71). Histology did not significantly impact OS (*P* = 0.73). After multivariable adjustment, histology did not have a significant effect on OS in the lobular group (HR = 0.59, 95% CI: 0.14–2.41; *P* = 0.46) or mixed group (HR = 0.58, 95% CI: 0.25–1.31; *P* = 0.19) compared with the ductal group (Table 3). S1 and S2 Figs show the DMFS and RFS by histology among patients in the M0 group.

### Outcomes in patients with metastatic disease

Among the 165 patients in the M1 group, metastatic disease progressed after initial diagnosis in 161. The 6-month TTP1 was 0.18 (95% CI: 0.12–0.24) (S3 Table). Histology did not significantly impact TTP1 (*P* = 0.84). After multivariable adjustment for age, race, hormone status, HER2 status, number of metastasis (continuous), brain metastasis, and visceral metastasis, histology did not have a significant effect on TTP1 in the lobular group (HR = 1.01, 95% CI: 0.53–1.94; *P* = 0.98) or mixed group (HR = 1.04, 95% CI: 0.57–1.88; *P* = 0.90) compared with the ductal group (S4 Table).

In the M1 group, 84 patients died. The 3-year OS estimate was 0.47 (95% CI: 0.38–0.56). Histology did not significantly impact OS (*P* = 0.71). After multivariable adjustment, histology did not have a significant effect on OS in the lobular group (HR = 1.20, 95% CI: 0.52–2.75; *P* = 0.66) or mixed group (HR = 0.91, 95% CI: 0.35–2.34; *P* = 0.84) compared with the ductal group (Table 3). S3 Fig show the TTP1 by histology among patients in the M1 group.

## Discussion

IBC is recognized with characteristic clinical features but not as with specific histologic subtype of breast cancer[1, 6, 8]. To the best of our knowledge, no study has previously reported the characteristics of lobular histology in IBC. Our study describes this entity for the first time. In this cohort of patients, they tended to be younger with a median age of diagnosis of 51 compared to non-inflammatory breast cancer patients where the median age of diagnosis is 61. A lobular histology was seen in 4.5% of IBC cases. This incidence is smaller than the proportion of ILC seen in conventional breast cancer[15].

The current therapeutic approach toward IBC is modeled after the treatment of locally advanced breast cancer [1], despite the fact that the clinical behavior of IBC and non-inflammatory locally advanced breast cancer have been shown to be distinct[4]. The differential effect of histology in non-inflammatory breast cancer is not only prognostic but has been shown to be predictive of response to chemotherapy[16,17]. In addition, it has been shown that conventional breast cancer subtypes such as hormone receptor and HER2 molecular subtypes have limited predictive and prognostic power in IBC[17,18]. Our patients with lobular histology were found had double the rates of metastatic disease, and despite controlling for multiple factors, histology did not have a significant effect on OS. Our finding that histology has no bearing on survival outcomes in patients with IBC, unlike that in n-IBC, further supports the hypothesis that IBC is a distinct both at the clinical level, in addition to the molecular level[9]. Bertucci et al. showed that discriminator genes between IBC and n-IBC are associated with cellular processes related to signal transduction, cell motility, adhesion, and angiogenesis[8]. Therefore, further investigation into the molecular profile of IBC is needed to delineate tumor biology and to explore the potential role of targeted agents against unique pathways in IBC[19]. For instance, in non-inflammatory lobular carcinomas E-cadherin loss is a defining feature and differentiates ILC from IDC[20]. Although, recent data has implicated that E-cadherin is involved in the oncogenic potential of IBC the differential effect in ductal and lobular histologic subtypes of IBC needs further elucidation[21]. While our lobular and mixed subtype was defined by histology without E-cadherin staining and there is the possibility of interobserver variability in assigning histology, there is no specific definition established in IBC for histological classification.

The histologic difference between lobular and ductal cancers in n-IBC is apparent at both clinical and molecular levels. In addition to gene expression analyses distinguishing lobular from ductal carcinomas[13, 14]. Cristofanilli et al. also showed that treatment effect did not contribute to the longer survival of ILC compared with IDC[11]. A meta-analysis comparing ILC and IDC in terms of pathological complete response rate (pCR) to neoadjuvant chemotherapy, IDC had a better pCR (16.7% for IDC vs. 5.9% for ILC; *P* < 0.00001)[22]. Thus, both clinical and genomic differences suggest an existing heterogeneity between IBC and n-IBC types [23]. Our finding therefore suggests that IBC is a distinct subtype of breast cancer driven by a unique molecular mechanism that overrides the heterogeneity imparted by histology. Moreover, this finding argues against including IBC under the same therapeutic umbrella as non-inflammatory breast cancer. Because IBC is underrepresented in studies involving breast cancer, results from such trials must be interpreted with caution until they are validated specifically in patients with IBC. Owing to the rarity of IBC, a concerted and committed multicenter strategy for designing therapeutic trials dedicated to IBC is needed.

We recognize the drawbacks of this study to include the inherent bias of a retrospective review. In addition, it should be noted that in 2010 the American Society of Clinical Oncology and College of American Pathologists issued guidelines of ER and PR status and recommended to consider the ER and PR assay positive if there is at least 1% positive tumor nuclei staining. However, our analysis spans several decades and ends prior to 2010. We thus determined ER and PR status by immunohistochemical staining, and this was considered positive if >10% of cells were stained.

Furthermore, despite a reasonable overall sample size the rate of lobular and mixed histologies were low. This may impact the statistical power of our analysis thus detecting any difference in survival between these groups becomes difficult.

## Conclusion

Lobular IBC is a rare but discrete histologic subtype of IBC. Nevertheless, our data suggests that there is no significant difference in the survival outcomes of patients with lobular IBC compared to those with ductal IBC. Therefore, due to the aggressive nature of IBC, present-day management of all patients with IBC must include an aggressive multidisciplinary approach composed of neoadjuvant multiagent chemotherapy, surgery, and radiation, irrespective of histology. However, the fact that IBC does not respect the histologic distinction characteristic of non-inflammatory breast cancer suggests that the IBC phenotype transcends the confines of histology. Future research into the molecular profile of IBC may help in understanding the biology of this unique clinicopathological phenotype of breast cancer.

## Supporting Information

S1 Fig Kaplan-Meier estimate of Distant Metastasis-Free Survival (DMFS) by histology among M0 patients. (TIF)

S2 Fig Kaplan-Meier estimate of Recurrence-Free Survival (RFS) by histology among M0 patients. (TIF)

S3 Fig Kaplan-Meier estimate of time to first progression (TTP1) by histology among M1 patients. (TIF)

S1 Table Survival estimates by patient and clinical characteristics among M0 patients. (DOCX)

S2 Table Cox proportional hazards models for distant metastasis-free survival (DMFS) and recurrence-free survival (RFS) among M0 patients. (DOCX)

S3 Table Survival estimates by patient and clinical characteristics among M1 patients. (DOCX)

S4 Table Cox proportional hazards models for time to first progression (TTP1) among M1 patients. (DOCX)
