# Supplementary figures and images for: Inflammatory Breast Cancer: A Distinct Clinicopathological Entity Transcending Histological Distinction
Source: PLoS One. 2016 Jan 11;11(1):e0145534. doi: 10.1371/journal.pone.0145534 (PMC4709074; doi:10.1371/journal.pone.0145534)

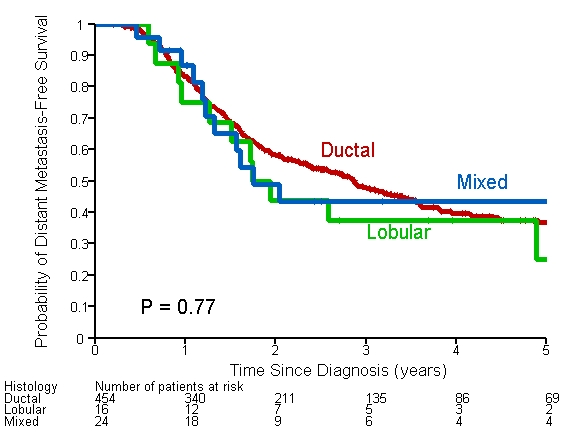

Supplement: S1 Fig — (TIF) [file pone.0145534.s001.tif]

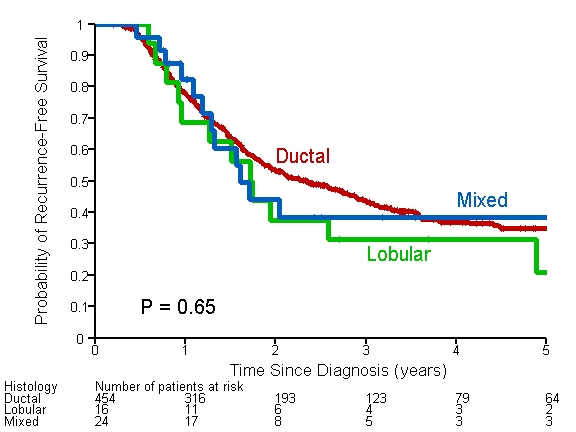

Supplement: S2 Fig — (TIF) [file pone.0145534.s002.tif]

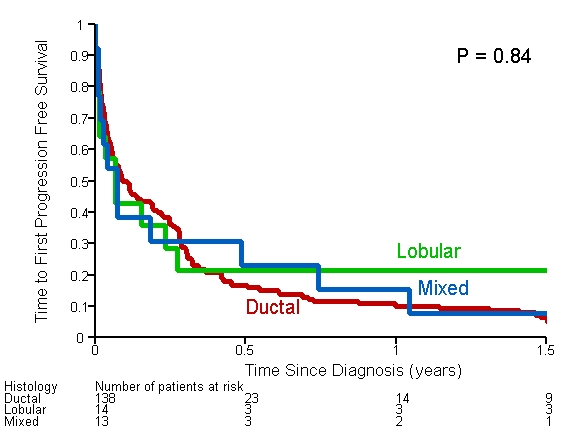

Supplement: S3 Fig — (TIF) [file pone.0145534.s003.tif]
