# Supplementary material for: Inflammatory Breast Cancer: A Distinct Clinicopathological Entity Transcending Histological Distinction
Source: PLoS One. 2016 Jan 11;11(1):e0145534. doi: 10.1371/journal.pone.0145534 (PMC4709074; doi:10.1371/journal.pone.0145534)
Supplement: S1 Table — (DOCX) [file pone.0145534.s004.docx]

**S1 Table: Survival estimates by patient and clinical characteristics among M0 patients**

|  | | **Distant Metastasis-Free Survival** | | | **Recurrence-Free Survival** | | |
| --- | --- | --- | --- | --- | --- | --- | --- |
|  | ***N* Patients** | ***N* Events** | **3-Year Estimate**  **(95% CI)** | ***P* value** | ***N* Events** | **3-Year Estimate**  **(95% CI)** | ***P* value** |
| Total | 494 | 263 | 0.48 (0.43, 0.52) |  | 278 | 0.43 (0.38, 0.48) |  |
| Histology |  |  |  |  |  |  |  |
| Ductal | 454 | 241 | 0.48 (0.43, 0.53) |  | 254 | 0.44 (0.39, 0.49) |  |
| Lobular | 16 | 11 | 0.37 (0.15, 0.59) |  | 12 | 0.31 (0.11, 0.53) |  |
| Mixed | 24 | 11 | 0.45 (0.22, 0.65) | 0.77 | 12 | 0.40 (0.18, 0.60) | 0.65 |
| Histology |  |  |  |  | . |  |  |
| Ductal | 454 | 241 | 0.48 (0.43, 0.53) |  | 254 | 0.44 (0.39, 0.49) |  |
| Lobular / Mixed | 40 | 22 | 0.41 (0.25, 0.57) | 0.68 | 24 | 0.36 (0.20, 0.51) | 0.52 |
| Age |  |  |  |  |  |  |  |
| ≤ 60 | 412 | 225 | 0.48 (0.42, 0.53) |  | 238 | 0.43 (0.38, 0.48) |  |
| > 60 | 82 | 38 | 0.48 (0.35, 0.60) | 0.97 | 40 | 0.44 (0.31, 0.56) | 0.86 |
| Menopausal Status |  |  |  |  |  |  |  |
| Pre | 230 | 124 | 0.44 (0.37, 0.51) |  | 134 | 0.39 (0.32, 0.46) |  |
| Post | 258 | 135 | 0.51 (0.44, 0.57) | 0.34 | 140 | 0.47 (0.40, 0.53) | 0.20 |
| Race |  |  |  |  |  |  |  |
| Non-Black | 450 | 232 | 0.50 (0.45, 0.55) |  | 247 | 0.45 (0.40, 0.50) |  |
| Black | 44 | 31 | 0.24 (0.12, 0.38) | 0.013 | 31 | 0.25 (0.13, 0.39) | 0.046 |
| Hormone Status |  |  |  |  |  |  |  |
| Negative | 223 | 126 | 0.40 (0.33, 0.47) |  | 134 | 0.37 (0.30, 0.43) |  |
| Positive | 242 | 115 | 0.58 (0.50, 0.64) | 0.003 | 122 | 0.51 (0.44, 0.58) | 0.002 |
| HER2 Status |  |  |  |  |  |  |  |
| Negative | 255 | 137 | 0.46 (0.39, 0.52) |  | 145 | 0.42 (0.36, 0.49) |  |
| Positive | 155 | 78 | 0.48 (0.39, 0.57) | 0.37 | 83 | 0.43 (0.34, 0.51) | 0.39 |
| Subtype |  |  |  |  |  |  |  |
| Hormone positive | 148 | 64 | 0.60 (0.51, 0.69) |  | 68 | 0.56 (0.47, 0.65) |  |
| HER2 positive | 155 | 78 | 0.48 (0.39, 0.57) |  | 83 | 0.43 (0.34, 0.51) |  |
| Triple negative | 105 | 71 | 0.27 (0.18, 0.36) | < 0.001 | 75 | 0.24 (0.16, 0.34) | < 0.001 |
| Grade |  |  |  |  |  |  |  |
| 1 or 2 | 109 | 55 | 0.55 (0.44, 0.65) |  | 57 | 0.52 (0.41, 0.62) |  |
| 3 | 358 | 190 | 0.46 (0.40, 0.52) | 0.15 | 202 | 0.41 (0.35, 0.47) | 0.08 |
| Clinical N |  |  |  |  |  |  |  |
| N0 | 68 | 39 | 0.52 (0.38, 0.63) |  | 41 | 0.45 (0.32, 0.57) |  |
| N1 | 241 | 123 | 0.52 (0.45, 0.59) |  | 131 | 0.46 (0.39, 0.53) |  |
| N2 | 53 | 35 | 0.39 (0.25, 0.52) |  | 36 | 0.37 (0.24, 0.51) |  |
| N3 | 123 | 59 | 0.43 (0.33, 0.53) | 0.30 | 62 | 0.41 (0.31, 0.50) | 0.66 |
| Lymphovascular Invasion |  |  |  |  |  |  |  |
| Negative | 166 | 67 | 0.61 (0.53, 0.69) |  | 69 | 0.59 (0.51, 0.67) |  |
| Positive | 294 | 176 | 0.42 (0.36, 0.48) | < 0.001 | 189 | 0.36 (0.30, 0.42) | < 0.001 |
| Adjuvant Chemo |  |  |  |  |  |  |  |
| No | 256 | 127 | 0.45 (0.38, 0.52) |  | 135 | 0.41 (0.34, 0.48) |  |
| Yes | 238 | 136 | 0.50 (0.43, 0.57) | 0.37 | 143 | 0.46 (0.39, 0.52) | 0.43 |
| Adjuvant Radiation |  |  |  |  |  |  |  |
| No | 129 | 81 | 0.31 (0.22, 0.40) |  | 87 | 0.27 (0.18, 0.36) |  |
| Yes | 365 | 182 | 0.53 (0.47, 0.59) | < 0.001 | 191 | 0.49 (0.43, 0.54) | < 0.001 |
