# Supplementary material for: Inflammatory Breast Cancer: A Distinct Clinicopathological Entity Transcending Histological Distinction
Source: PLoS One. 2016 Jan 11;11(1):e0145534. doi: 10.1371/journal.pone.0145534 (PMC4709074; doi:10.1371/journal.pone.0145534)
Supplement: S2 Table — (DOCX) [file pone.0145534.s005.docx]

**S2 Table: Cox proportional hazards models for distant metastasis-free survival (DMFS) and recurrence-free survival (RFS) among M0 patients**

|  | Distant Metastasis-Free Survival | | | Recurrence-Free Survival | | |
| --- | --- | --- | --- | --- | --- | --- |
|  | **HR** | **95% CI** | ***P* value** | **HR** | **95% CI** | ***P* value** |
| Lobular vs. Ductal | 1.62 | 0.75 to 3.51 | 0.22 | 1.39 | 0.64 to 3.00 | 0.40 |
| Mixed vs. Ductal | 0.84 | 0.43 to 1.65 | 0.62 | 0.86 | 0.45 to 1.63 | 0.64 |
| Age: > 60 vs. ≤ 60 | 0.95 | 0.64 to 1.42 | 0.81 | 0.78 | 0.53 to 1.16 | 0.22 |
| Race: Black vs. Non-black | 1.29 | 0.80 to 2.07 | 0.29 | 1.15 | 0.72 to 1.85 | 0.56 |
| Hormone status: Positive vs. Negative | 0.53 | 0.39 to 0.73 | < 0.0001 | 0.50 | 0.37 to 0.67 | < 0.0001 |
| HER2 status: Positive vs. Negative | 0.69 | 0.51 to 0.94 | 0.017 | 0.69 | 0.51 to 0.93 | 0.015 |
| Lymphovascular invasion: Yes vs. No | 1.91 | 1.39 to 2.63 | < 0.0001 | 2.26 | 1.66 to 3.09 | < 0.0001 |
| Adjuvant Radiation: Yes vs. No | 0.45 | 0.33 to 0.63 | < 0.0001 | 0.39 | 0.28 to 0.53 | < 0.0001 |
