# Supplementary material for: Inflammatory Breast Cancer: A Distinct Clinicopathological Entity Transcending Histological Distinction
Source: PLoS One. 2016 Jan 11;11(1):e0145534. doi: 10.1371/journal.pone.0145534 (PMC4709074; doi:10.1371/journal.pone.0145534)
Supplement: S3 Table — (DOCX) [file pone.0145534.s006.docx]

**S3 Table: Survival estimates by patient and clinical characteristics among M1 patients**

|  |  | **Time To 1^st^ Progression** | | |
| --- | --- | --- | --- | --- |
|  | ***N* Patients** | ***N* Events** | **6-Month Estimate**  **(95% CI)** | ***P*** |
| Total | 165 | 161 | 0.18 (0.12, 0.24) |  |
| Histology |  |  |  |  |
| Ductal | 138 | 135 | 0.17 (0.11, 0.23) |  |
| Lobular | 14 | 13 | 0.21 (0.05, 0.45) |  |
| Mixed | 13 | 13 | 0.23 (0.06, 0.47) | 0.84 |
| Histology |  |  |  |  |
| Ductal | 138 | 135 | 0.17 (0.11, 0.23) |  |
| Lobular / Mixed | 27 | 26 | 0.22 (0.09, 0.39) | 0.74 |
| Age |  |  |  |  |
| ≤ 60 | 126 | 122 | 0.17 (0.11, 0.25) |  |
| > 60 | 39 | 39 | 0.18 (0.08, 0.31) | 0.53 |
| Menopausal Status |  |  |  |  |
| Pre | 75 | 72 | 0.17 (0.10, 0.27) |  |
| Post | 88 | 87 | 0.17 (0.10, 0.26) | 0.24 |
| Race |  |  |  |  |
| Non-Black | 134 | 132 | 0.17 (0.10, 0.27) |  |
| Black | 31 | 29 | 0.17 (0.10, 0.26) | 0.03 |
| Hormone Status |  |  |  |  |
| Negative | 69 | 68 | 0.13 (0.06, 0.22) |  |
| Positive | 86 | 86 | 0.17 (0.10, 0.26) | 0.59 |
| HER2 Status |  |  |  |  |
| Negative | 111 | 111 | 0.14 (0.08, 0.21) |  |
| Positive | 41 | 41 | 0.12 (0.04, 0.24) | 0.20 |
| Subtype |  |  |  |  |
| Hormone positive | 71 | 71 | 0.18 (0.10, 0.28) |  |
| HER2 positive | 41 | 41 | 0.12 (0.04, 0.24) |  |
| Triple negative | 40 | 40 | 0.05 (0.01, 0.15) | 0.06 |
| Grade |  |  |  |  |
| 1 or 2 | 39 | 38 | 0.31 (0.17, 0.45) |  |
| 3 | 114 | 111 | 0.12 (0.07, 0.19) | 0.20 |
| Lymphovascular Invasion |  |  |  |  |
| Negative | 34 | 32 | 0.15 (0.05, 0.28) |  |
| Positive | 74 | 72 | 0.15 (0.08, 0.24) | 0.70 |
| Number of Metastasis |  |  |  |  |
| 0 | 9 | 5 | 0.44 (0.14, 0.72) |  |
| 1 | 82 | 82 | 0.13 (0.07, 0.22) |  |
| 2+ | 74 | 74 | 0.19 (0.11, 0.29) | 0.001 |
| Brain, Meninges, Spinal Cord Metastasis |  |  |  |  |
| No | 161 | 157 | 0.17 (0.12, 0.24) |  |
| Yes | 4 | 4 | 0.25 (0.01, 0.67) | 0.84 |
| Visceral Metastasis |  |  |  |  |
| No | 91 | 87 | 0.16 (0.10, 0.25) |  |
| Yes | 74 | 74 | 0.19 (0.11, 0.29) | 0.65 |
