# Supplementary material for: Inflammatory Breast Cancer: A Distinct Clinicopathological Entity Transcending Histological Distinction
Source: PLoS One. 2016 Jan 11;11(1):e0145534. doi: 10.1371/journal.pone.0145534 (PMC4709074; doi:10.1371/journal.pone.0145534)
Supplement: S4 Table — (DOCX) [file pone.0145534.s007.docx]

**S4 Table: Cox proportional hazards models for time to first progression (TTP1) among M1 patients**

|  | Time to First Progression | | |
| --- | --- | --- | --- |
|  | HR | 95% CI | *P* |
| Lobular vs. Ductal | 1.01 | 0.53 to 1.94 | 0.98 |
| Mixed vs. Ductal | 1.04 | 0.57 to 1.88 | 0.90 |
| Age: > 60 vs. ≤ 60 | 0.65 | 0.44 to 0.97 | 0.036 |
| Race: Black vs. Non-black | 0.64 | 0.42 to 1.00 | 0.05 |
| Hormone status: Positive vs. Negative | 0.69 | 0.48 to 0.99 | 0.045 |
| HER2 status: Positive vs. Negative | 1.12 | 0.75 to 1.69 | 0.57 |
| Number of metastasis (continuous) | 0.96 | 0.86 to 1.08 | 0.53 |
| Brain metastasis: Yes vs. No | 1.07 | 0.38 to 3.03 | 0.90 |
| Visceral metastasis: Yes vs. No | 0.71 | 0.49 to 1.03 | 0.07 |
